# Twenty-one Years of Undergraduate Medical Student Research Training at the University of the Free State (UFS), South Africa

**DOI:** 10.1007/s40670-024-02107-8

**Published:** 2024-07-03

**Authors:** Gina Joubert, Wilhelm J. Steinberg, Francois C. van Rooyen

**Affiliations:** 1 Department of Biostatistics, School of Biomedical Sciences, Faculty of Health Sciences, University of the Free State, 205 Nelson Mandela Drive, Bloemfontein, 9300 South Africa; 2 Department of Family Medicine, School of Clinical Medicine, Faculty of Health Sciences, University of the Free State, Bloemfontein, South Africa

**Keywords:** Undergraduate, Medical students, Research, Training, Experience, Outcomes, Publications

## Abstract

**Introduction:**

Since 2001, undergraduate medical students at the University of the Free State (UFS), South Africa, plan, perform, and report on their research projects during semesters 2–5 of their ten-semester training. We describe the research modules and review the projects of the first 21 years.

**Methods:**

This cohort study included all undergraduate medical student projects that formed part of the first 21 presentations of the research modules. Information was obtained from material archived by the module leaders. Students’ 2020 feedback on the modules was summarised.

**Results:**

In total, 607 projects were planned (range 22–35 per year) and involved 229 supervisors. Only four projects were not completed. Thirty-nine Faculty departments/divisions/units provided supervision with Family Medicine, Internal Medicine, and Paediatrics and Child Health each supervising 60 or more groups. Projects were predominantly quantitative (99.7%); only 4.9% of projects involved an intervention or experiment. Main topics were infectious diseases (10.5%), mental health (8.9%), and cancer (8.7%). Data subjects were mainly patients (61.9%) and undergraduate students (12.0%), and data collection was mostly performed at the faculty’s training hospitals or laboratories (71.4%). The most positive aspect indicated by students was the exposure to and learning about research; the most negative aspects were group work and supervisors.

**Conclusion:**

The projects received support from a broad spectrum of supervisors and covered a wide variety of topics. Given the timing of the projects in the training programme, the mainly quantitative and observational nature of the projects was appropriate. Attention to supervision and group work is required.

## Introduction

In an era of evidence-based medicine and continuing professional development, research skills are essential tools for medical practitioners. As stipulated by the Health Professions Council of South Africa (HPCSA) [1], “Scholar” is one of the core competencies for undergraduate students in medical teaching and learning programmes in South Africa. Furthermore, the HPCSA requires that “healthcare practitioners demonstrate a lifelong commitment to reflective learning, as well as the creation, dissemination, application and translation of knowledge”. Other stated core competencies such as “Health Advocate”, “Manager”, and “Communicator” [1] can also benefit from research skills. Internationally, undergraduate medical student exposure to research has been promoted as the solution to increase the declining numbers of “clinician-scientists” [2, 3] and medical academics [4].

In South Africa, all medical schools include some aspects of an undergraduate medical student research project, with various approaches being used, such as the project being an elective [5], performed in a community-based setting [6], or a protocol only needing to be developed [7]. Of the ten medical schools in South Africa, the University of the Free State (UFS) was one of the first two medical schools to introduce a compulsory research project in the training programme [8].

In the previous 6-year undergraduate medical programme at the UFS (the final class graduated in 2004), students received lectures on epidemiology and biostatistics during their third year. In their final year of training, students could choose to do a research project or do case studies as part of the 8-week Obstetrics and Gynaecology training block. In 2000, with the introduction of the current 5-year undergraduate medical programme, compulsory research projects were included in the curriculum as part of the pre-clinical training. In 2022, the 21st year group of undergraduate medical students completed their research modules.

Undergraduate medical student feedback regarding involvement and interest in research has been largely positive. In a systematic review and meta-analysis that included studies from the USA, Canada, UK, China, Croatia, Malaysia, the Netherlands, Norway, and Pakistan, Naing et al. [9] reported that 74% (95% confidence interval (CI), 60.8–87.6%) of students who had been involved in research had positive attitudes towards their research experience. Nel et al. [10] reported that 61% of medical students sampled at the University of Cape Town (UCT) had a positive attitude towards research and 74% indicated that they considered it important for their training to participate in research. At the time of the study by Nel et al., the exposure of medical students at UCT to research was limited [10]. At the University of KwaZulu-Natal (UKZN), responses were even more positive among students who had completed their compulsory research modules, indicating that they had enjoyed learning about research (78%) and that the training activities enhanced their understanding of the research process (84%) [11].

## Aim

The aim of this study was to give an overview of the first 21 rounds (2002–2022) of undergraduate medical student research in the School of Medicine, UFS, in terms of:

- The module approach;
- The number of projects;
- The number of supervisors and departments involved;
- The characteristics of projects in terms of setting, topic and methodology;
- Publication success; and
- Student feedback.

In addition, trends over time (in 7-year intervals 2002–2008, 2009–2015, 2016–2022) were summarised, and associations between project characteristics, publication success, and winning the module prize (awarded for the highest marks for the presentation and report combined) were investigated. Such a review not only provides an opportunity for reflection for the institution itself but can also give insight to other institutions regarding approaches to consider and challenges to expect.

## Methods

This retrospective cohort study included all undergraduate medical student projects that formed part of the first 21 completed rounds of the Epidemiology, Biostatistics, and Research Project modules. Information was obtained from module material archived by the module leaders, in particular the Excel archive list of module projects, as well as hard copy or electronic versions of final project protocols and reports. Details regarding publication status were checked with the Faculty medical editors. Annually, at the project presentations in semester 5 (the last formal contact session), students were invited to complete anonymous module evaluation forms consisting of a number of closed questions and three open-ended questions regarding the positive and negative aspects of the modules and any suggestions the students may have. Due to COVID-19 restrictions regarding attendance at these sessions, module evaluations were not conducted in 2021 and 2022. The responses of 2020 as the most recent available information commenting on the modules as they are currently presented were summarised. Students’ open responses were categorised into themes by means of discussion between the first two authors (GJ and WJS).

The protocol for the current study was approved by the UFS Health Sciences Research Ethics Committee (HSREC; reference number UFS-HSD2022/0164/3105). No student or supervisor names are reported. No individual projects are reported in an identifiable way. Approval was also obtained from UFS authorities.

The 63 projects completed in 2002 and 2012 were included in a pilot study to test the methodology of data collection. No changes were necessary and pilot study cases were included in the main study.

The data were captured in an Excel (version 2016) spreadsheet and analysed using SAS Version 9.4 (SAS Institute Inc.; Cary NC, USA), mainly in the form of frequencies and percentages. Associations between variables were investigated using chi-squared or Fisher’s exact tests, with *p* < 0.05 considered statistically significant.

## Results

### Research Modules

During the second and third semesters of the ten-semester medical training programme at the UFS, students receive lectures and assessments on epidemiology, biostatistics, and the research process (Table 1). In semesters 2 to 5, students (in small groups established by themselves) go through the research process, from protocol planning and ethics submission to reporting their research findings, under the guidance of a medical or scientist supervisor from a clinical or laboratory discipline. Supervisors take part on a voluntary basis and student project groups are allocated to a supervisor in a specific field based on the preference for a study area indicated by the project group. Project groups and supervisors (ideally) discuss potential research ideas to finalise the topic. Module leaders monitor progress throughout and provide support when required.

**Table 1:** Undergraduate medical student research projects: main activities per semester

Semester	Activity	Primary responsible persons
Semester 2	Introduction to research and protocol writing	Module team*
	Lectures and assessments on epidemiology and research methodology	Module team
	Allocation of project groups to supervisors	Module team
	Finalisation of the research topic	Students and supervisors
Semester 3	Lectures and assessments on biostatistics	Module team
	Protocol finalisation	Students and supervisors
	Protocol submission to the Health Sciences Research Ethics Committee	Students
Semester 4	Data collection	Students
Semester 5	Project completion	Students
	Data analysis	Biostatisticians
	Group presentation and submission of the report	Students

^*^The module team consists of biostatisticians and clinicians

In the initial years (with class sizes of 70 to 90), students could work on projects individually or in pairs but with increasing class sizes, the number of students per project group was increased to 3 to 4, and is currently 6 to 8 students (current class size 160 to 180 students). To enhance project viability, students who have to repeat an academic year but have passed the research modules continue with their projects as planned. Group marks are allocated for the protocol, presentation and final report. Students may choose to write individual reports. At the project presentations (where one speaker chosen by the group presents on behalf of the group), the module team acts as adjudication panel and students assess the presentation of other project groups.

The module team has annual planning sessions to discuss student feedback and module issues and consider possible changes to the modules. Refinements that have been implemented over time when the need was identified by the module team included:

- Clarifying the role and responsibilities of supervisors to try to ensure some uniformity regarding supervision approaches;
- Encouraging supervisors to attend project discussion sessions between project groups and the module team to keep all involved in the project informed;
- Clarifying expectations and guidelines for students and supervisors regarding the format, content and assessment of the protocol, presentation and report to ensure uniformity;
- A set amount available from the School of Medicine Research Committee for project funding to prevent costs from hampering some students’ ability to perform their project;
- A formal session where student groups present their project topics to the module team and supervisors early in the planning phase to ensure that all involved have an early opportunity to comment on and guide the planning;
- Supervisors keeping a register of attendance of meetings with the project group and the module team subtracting marks for students for non-attendance to promote student participation;
- Allocating marks for draft protocols that project groups submit to the module team for feedback to ensure that students apply their minds and do not rely only on the feedback;
- Providing general feedback online to the class after the project presentations to enable changes to the project reports that are submitted 2–4 weeks later;
- Expecting students to write individual abstracts and conclusions for the research report to incorporate some individual work in what mainly is a group activity; and
- Increasing practical sessions regarding administrative aspects of, for example, the ethics application and documentation to ensure that these aspects do not cause time delays and uncertainty.

Changes that have been attempted over the years but abandoned included the following:

- A block set aside in the programme for students to perform data collection—it was found that not all projects allowed for data collection in the same time period.
- Students assessing group member activity and group marks adjusted accordingly for individual students—it was found that group members varied in their assessment and there were frequently mitigating circumstances why a specific group member did not participate at certain stages of the research process.
- Discussion meetings of all supervisors with the module team—finding appropriate time slots was difficult. Supervisors are reminded and encouraged to contact module leaders as needed.
- Formal written feedback from supervisors regarding their experiences—most supervisors preferred to give informal feedback when they happened to see the module leaders in the corridors.
- A feedback session with the class after reports had been marked—this was usually close to exam time and it was difficult to find an appropriate slot on the semester planner. Project groups can, however, contact the module leaders for feedback.

### Projects

**Fig. 1:**
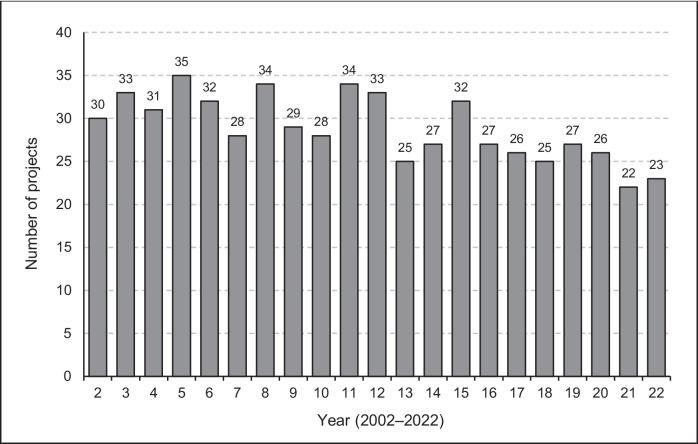
Number of undergraduate medical student projects annually (*n* = 607)

During the study period, 607 projects were planned with the number per year ranging from 22 (in 2021) to 35 (in 2005), as shown in Fig. 1.

Thirty-nine faculty departments/divisions/units were involved in supervision; the top five in terms of the number of groups supervised were the Departments of Family Medicine (86 project groups), Paediatrics and Child Health (66), Internal Medicine (64), Obstetrics and Gynaecology (47), and Psychiatry (44). These supervisors were all medical clinicians or clinical psychologists. Three departments, namely Family Medicine, Paediatrics and Child Health, and Internal Medicine, were in the top five in each of the three time periods, with Family Medicine supervising 23.9% (42/176) of the projects in the period 2016–2022.

Two hundred and twenty-nine supervisors (of whom six were in private practice) guided students, with nine supervisors guiding at least ten groups. One supervisor guided 19 groups (one each in 19 of the 21 year groups). Fourteen supervisors had completed these undergraduate research modules as part of their own undergraduate training. For 17 (2.8%) projects, supervisors preferred to work in a team of two (15 projects) or three (two projects) co-supervisors. For 24 (4.0%) projects, the supervisor was guiding more than one project group in the same year group. For 15 (2.5%) projects, supervisors needed to be replaced due to moving away (*n* = 7) or not fulfilling their supervisory duties (*n* = 8).

Given the wide range of departments involved in supervision, the research themes covered were similarly wide, with infectious conditions and their treatment (10.5%), mental health (8.9%), and cancer (8.7%) being the most common.

Projects were predominantly quantitative (99.7%; Table 2) and only 30 projects (4.9%) involved an intervention or experiment. Approximately half the projects (50.1%) consisted of an audit, determined the profile of a group, or determined an incidence or prevalence, and 10.5% investigated one or more aspects of knowledge, attitudes, and practices. Similar percentages of projects used prospective and retrospective data collection, with no marked change over the years. Projects that won the module prize were more likely to have used prospective data collection (15/24; 62.5%). Data were mainly collected regarding patients (61.9%) and in one of the faculty’s training hospitals (primary, secondary, and tertiary healthcare settings, including outpatients and specialist clinics) or laboratories (71.4%). A significant increase in the percentage of projects collecting data about undergraduate students occurred over time: 9.0% in 2002–2008, 11.1% in 2009–2015 and 17.1% in 2016–2022 (*p* = 0.03). The percentage of projects collecting data about patients declined from 65.3% in 2002–2008 to 55.1% in 2016–2022.

**Table 2:** Undergraduate medical student research projects’ characteristics and outcomes

	Total (*N* = 607)	2002–2008 (*n* = 223)	2009–2015 (*n* = 208)	2016–2022 (*n* = 176)
	*n* (%)	*n* (%)	*n* (%)	*n* (%)
*Study design*				
Quantitative	605 (99.7)	223 (100)	206 (99.0)	176 (100)
Qualitative	2 (0.3)	0 (0)	2 (1.0)	0 (0)
Experiment/intervention	30 (4.9)	12 (5.4)	11 (5.3)	7 (4.0)
*Data collection*				
Prospective	307 (50.6)	107 (48.0)	111 (53.4)	89 (50.6)
Retrospective	295 (48.6)	115 (51.6)	95 (45.7)	85 (48.3)
Both	5 (0.8)	1 (0.5)	2 (1.0)	2 (1.1)
Data collection subjects*	**(*n* = 606)**	**(*n* = 222)**	**(*n* = 208)**	**(*n* = 176)**
Patients	375 (61.9)	145 (65.3)	133 (63.9)	97 (55.1)
Undergraduate students	73 (12.0)	20 (9.0)	23 (11.1)	30 (17.1)
Health workers	57 (9.4)	18 (8.1)	18 (8.7)	21 (11.9)
Parents/caregivers	28 (4.6)	13 (5.9)	5 (2.4)	10 (5.7)
Data collection site*	**(*n* = 597)**	**(*n* = 217)**	**(*n* = 205)**	**(*n* = 175)**
Faculty’s training hospitals or laboratories	426 (71.4)	154 (71.0)	146 (71.2)	126 (72.0)
Other healthcare setting (clinic, private hospital)	75 (12.6)	34 (15.7)	26 (12.7)	15 (8.6)
UFS campus (classrooms/residences/cafeteria)	73 (12.2)	19 (8.8)	22 (10.7)	32 (18.3)
*Project completed*				
Yes	603 (99.3)	222 (99.6)	208 (100)	173 (98.3)
No	4 (0.7)	1 (0.4)	0 (0)	3 (1.7)
*Project published*				
Yes	136 (22.4)	42 (18.8)	38 (18.3)	56 (31.8)
No	471 (77.6)	181 (81.2)	170 (81.7)	120 (68.2)

^*^More than one option could apply per project, and only the main categories are reported

Only four projects (0.7%; Table 2) were not completed, i.e. did not go through all the steps of planning, data collection, and reporting. The reasons were as follows: students in the group all failed out of the medical training programme before completion of the research modules (one group), the project was no longer feasible after the supervisor left the country (one group), and the protocol was never finalised to the requirements of the Ethics Committee (two groups).

Approximately one in five projects was published (22.4%). The percentages of published projects increased from 18.8% and 18.3% for projects in the time periods 2002–2008 and 2009–2015, respectively, to 31.8% for projects in the period 2016–2022 (a small number of projects from the latter time period are currently in submission or being prepared for submission). Two projects led to two publications each, resulting in a total of 138 publications, mainly in accredited peer-reviewed journals (*n* = 137; 99.3%), as full-length manuscripts (*n* = 123; 89.1%) and with student group members as authors (*n* = 135; 97.8%). Of the prize-winning projects, 45.8% (11/24) were published. Prospective studies were more likely to be published (78/307; 25.4%) than retrospective studies (57/295; 19.3%). Only two (6.7%) of the 30 studies that involved an experiment were published.

### Student Feedback

Of the 155 students involved in projects that were finalised in 2020, 103 (66.4%) responded to one or more of the open-ended questions. Of these, 101 (98.1%) made positive comments and 58 (56.3%) negative comments regarding the research projects. The most common themes are indicated in Table 3. Regarding group work as a negative aspect, some student comments included “participation was sometimes a problem – some members were not fully involved”, “poor communication between group members”, and “group dynamics are always difficult”. However, other students stated a positive experience in this regard, such as “enjoyed working in group!”, “groups made the modules easier”, and “great teamwork and comradery (sic) is learned”. The negative aspect of supervisors related to availability and involvement evoked the following responses: “difficulty getting to meet our study leader – he was very busy; would have appreciated more support from our study leader”, “our study leader wasn’t very involved and didn't always know what was going on in our study”, “the study leader wasn’t available when help or guidance was needed”, “study leaders that are not committed to the project”, “the study leader availability was a problem and cooperation we were quite alone most of the project with module leaders almost giving better guidance”. However, comments such as “the fact that study leaders as well as module heads and members are so involved helped ensure projects are finished on time and that we actually knew what was happening and what we had to do” and “our study leader was also excellent” were also made.

**Table 3:** Most common themes emanating from students’ open responses regarding research projects—2020 module evaluation

Positive aspects (number of respondents)	Negative aspects (number of respondents)
1. Exposure to and learning about research (55)	1. Group work (17) 1. Supervisors (17)
2. Support from module team (25)	2. Organisation/guidelines (13)
3. Organisation/guidelines (18)	3. Time/timing (11)
4. Exposure to medical field/environment (12)	4. Ethics and other approval delays (8)
5. Group work (10)	

Forty-eight students (46.6% of those completing the evaluation forms) made some suggestions, mainly regarding organisation/guidelines (11 respondents), supervisors (10 respondents), and module time or timing (9 respondents).

More than 80% (129; 83.2%) of students completing their projects in 2020 responded to at least some of the closed-ended questions. Their responses are summarised in Table 4. More than 70% of responding students indicated that the time was sufficient, that supervisors were available and gave good guidance, and that the modules were informative. Only 59.4% of students agreed that the relevance of the modules to the practice of medicine was clear and 65.9% that the modules were essential in the programme, with approximately 30% of students being unsure.

**Table 4:** Student responses to closed questions regarding research modules—2020 module evaluation

	Disagree	Unsure	Agree
	*n* (%)	*n* (%)	*n* (%)
The relevance of the modules to the practice of medicine is clear (*n* = 128)	13 (10.2)	39 (30.5)	76 (59.4)
Overall, the time available was sufficient for mastering the content of the modules (*n* = 125)	5 (4.0)	23 (18.4)	97 (77.6)
The supervisor gave good guidance (*n* = 129)	14 (10.9)	24 (18.6)	91 (70.5)
The supervisor was available (*n* = 129)	17 (13.2)	18 (14.0)	94 (72.9)
The modules were interesting (*n* = 129)	20 (15.5)	58 (45.0)	51 (39.5)
The modules were informative (*n* = 129)	6 (4.7)	29 (22.5)	94 (72.9)
The modules are essential in the learning programme (*n* = 129)	9 (7.0)	35 (27.1)	85 (65.9)
The modules fulfilled my expectations (*n* = 129)	11 (8.5)	49 (38.0)	69 (53.5)

## Discussion

Marais et al. [5] stated that undergraduate students’ research engagement “might be enhanced if levels of choice are structured into the curriculum such that students’ needs for autonomy, competence, and relatedness are met”. In our programme, the research project is compulsory but some choice is given, namely the self-formation of project groups and choices regarding the study field in which student groups wish to do a project. The projects mostly represent a combination of the apprenticeship model (working closely with a supervisor) and the inquiry project model (students play a role in topic selection) outlined by Zimbardi and Myatt [12].

Ommering et al. [13] emphasised the importance of providing “an experiential opportunity by involving students in every stage of the scientific research process”. The extended period available for the research projects in our programme (approximately 18 months from first project ideas to submission of the final project report) ensures that ample time is available for all aspects of the project, and allows for waiting periods for HSREC and authority approval. It is, however, at times difficult to motivate students and supervisors to keep momentum over this extended period. Clear timelines and guidelines for the various phases of the research process are crucial to ensure that all groups complete the research process successfully. Furthermore, the module team needs adaptability and problem-solving skills to adjust to, for example, fluctuating class sizes and supervisor availability.

Different project groups experience the research process differently due to project group dynamics, different approaches by different supervisors (some of them inexperienced researchers), and different challenges posed by different research projects and research settings. As a reflection of this, the theme “Group work” was among the top five of both positive and negative aspects indicated by students in open responses in 2020.

Mass-Hernández et al. [14], in their review of the literature regarding problems, solutions, and outcomes of undergraduate research in medicine, concluded that not only should research training and exposure be included in the curriculum, but also “students need to have adequate collaboration and guidance from experienced researchers”. In the realist review of Cornett et al. [15], adequately supported and structured experiences and quality supervision were found to contribute to successful outcomes. In our modules, the continuous active involvement of the module team ensures a measure of standardisation. The fact that the module team consists of biostatisticians and clinicians ensures wide-ranging support. Module team support was the second most frequently mentioned positive aspect in students’ open responses in 2020. This support should be extended to cover final feedback to groups regarding their presentation and report to ensure that students achieve maximum benefit from all stages of the research process.

As seen from the large number of departments/units/divisions providing supervision, there was broad support for these research modules from the faculty. Departmental involvement did, however, fluctuate over time due to specific staffing challenges in certain departments. There was also faculty understanding and support for the fact that these modules are different in nature from most other modules in the programme, and approval was given that students who have to repeat a year but pass the research modules continue with their projects. The research modules are the only modules in the programme to which this arrangement is applicable.

The vast majority of students were exposed to the whole research process from planning to reporting, with nearly all projects completed. It was challenging to accommodate students whose supervisors left during the course of the project, students who could not finalise their protocol to the satisfaction of the ethics committee, students who were removed from groups due to requests by the supervisor, other group members or parents, students who interrupted their studies for an extended period, and students who failed the 6-year programme and subsequently entered the 5-year programme. Such students were usually incorporated into another group or, in a few isolated cases, were given an already existing dataset to report on.

Most projects were quantitative observational, which seems appropriate for a first exposure to the research process. Many projects were done in a setting within the Free State Province healthcare environment and dealt with patient or health worker information. Such exposure adds value to the training of students in the pre-clinical phase of their programme. An increase in the percentage of projects involving students as participants was seen over the 21 years. This has also been observed in other applications to the local institutional Ethics Committee [16]. As cautioned by Joubert et al. [16], topics should not be chosen specifically so that students, a potentially vulnerable population that can be reached fairly easily, are the participants. UFS authorities have refused permission for some medical student projects dealing with student populations, although the protocol had been conditionally approved by the Ethics Committee. This is a further challenge for the module team to address.

Publication success increased markedly over the years. Publication is not a stated outcome of the modules but gives recognition to students and supervisors. We request students to write a full project report rather than an article manuscript, attempting to ensure that all information required for a subsequent publication is available. The requirements of a research report of students new to research and the specific subject matter are different from those of a publishable manuscript [17]. The faculty medical editors played a substantial role in assisting with adapting student reports to publishable manuscripts, especially in the period 2016–2022. Our publication figure for the period 2016–2022 (31.8%) compares favourably with recently reported percentages of medical students publishing in Peru (14%) [18], Colombia (17%) [19], and the Netherlands (27.7%) [20], and is similar to the publication rate of the Bachelor of Medical Sciences (BMedSc) Honours theses (32.7%) at the University of Otago Medical School, New Zealand [21].

The open responses received from the students in 2020 were similar to those received from the first four year groups completing the research projects [22]. At that stage, the main negative feedback was about the availability and commitment of supervisors, and this aspect was still raised by students in 2020, despite subsequent changes to provide supervisors with more comprehensive guidelines. Delport et al. [7] reported that surveys and focus groups of University of Pretoria students identified the lack of choice regarding research topics, uncommitted group members, and supervisors as main negative factors. The assessment of the contribution of individual students to group activities remains challenging in our modules, especially with the increasing size of project groups. Some compulsory individual work has been built into the report writing and student attendance of meetings is assessed but these are minimal attempts at gauging individual inputs.

In 2006, the most common positive themes mentioned by UFS students were the exposure to research, the organisation of the modules, and acquiring general skills [22]. It is interesting to note that in 2020, “Organisation and guidelines”, which are the same for all students, were perceived by some students as a positive aspect and others as a negative aspect. As seen from the students’ responses to closed-ended questions in 2020, despite numerous positive comments, some students still need to be convinced that the research component is essential in their packed academic programme.

## Conclusion

At our institution, adaptability by the module team was necessary to address challenges encountered. The undergraduate medical student projects received support from a broad spectrum of supervisors and covered a wide variety of topics. Given the timing of the projects in the students’ training programme, it was appropriate that projects were mainly quantitative observational. The students’ exposure to the health environment potentially added value to their training. Students were positive regarding their exposure to research but students’ open responses indicated that problems related to supervision and group work need further attention. The findings at our institution can guide other institutions in approaches to consider.

## Recommendations

In general,

- Staff of such modules or programmes (which should ideally include biostatisticians and clinicians) must be committed and work closely together to solve problems as they arise and to revise the modules where necessary.
- The approach of exposing students to experiential learning as the basis for their research knowledge over an extended period of time should be followed.

At our institution,

- Challenges regarding group work and supervision require further action.
- Formal feedback to project groups regarding their presentations and final reports need to be implemented to ensure students benefit from all aspects of the research process.

## Data Availability

Data are available from the corresponding author upon reasonable request.

## References

[1] Health Professions Council of South Africa (HPCSA). Core competencies for undergraduate students in clinical associate, dentistry and medical teaching and learning programmes in South Africa. Pretoria: Health Professions Council of South Africa; 2014. https://www.hpcsa-blogs.co.za/wp-content/uploads/2017/04/MDB-Core-Competencies-ENGLISH-FINAL-2014.pdf. Accessed 14 February 2024.

[2] Ommering BWC, Van Blankenstein FM, Waaijer CJF, Dekker FW. Future physician-scientists: could we catch them young? Factors influencing intrinsic and extrinsic motivation for research among first-year medical students. Perspect Med Educ. 2018;7(4):248–55. 10.1007/S40037-018-0440-Y. 30006870 10.1007/s40037-018-0440-y PMC6086821

[3] Epstein N, Fischer MR. Academic career intentions in the life sciences: can research self-efficacy beliefs explain low numbers of aspiring physician and female scientists? PLoS ONE. 2017;12(9):e0184543. 10.1371/journal.pone.0184543. 28910334 10.1371/journal.pone.0184543 PMC5598975

[4] Jacobsen GW, Ræder H, Stien MH, Munthe LA, Skogen V. Springboard to an academic career – a national medical student research program. PLoS ONE. 2018;13(4):e0195527. 10.1371/journal.pone.0195527. 29708980 10.1371/journal.pone.0195527 PMC5927424

[5] Marais DL, Kotlowitz J, Willems B, Barsdorf NW, Van Schalkwyk S. Perceived enablers and constraints of motivation to conduct undergraduate research in a Faculty of Medicine and Health Sciences: what role does choice play? PLoS ONE. 2019;14(3):e0212873. 10.1371/journal.pone.0212873. 30865658 10.1371/journal.pone.0212873 PMC6415790

[6] Knight SE, Van Wyk JM, Mahomed S. Teaching research: a programme to develop research capacity in undergraduate medical students at the University of KwaZulu-Natal, South Africa. BMC Med Educ. 2016;16(1):61. 10.1186/s12909-016-0567-7. 26879830 10.1186/s12909-016-0567-7 PMC4754994

[7] Delport R, Dreyer A, Maart R, MohamedSharif A, Nekaka R, Turner A, Wolvaardt J. Undergraduate research – a tale of three African institutions. Afr Health Sci. 2023;23(2):743–52. 10.4314/ahs.v23i2.85. 38223607 10.4314/ahs.v23i2.85 PMC10782332

[8] Dommisse J, Joubert G. Profile of research methodology and statistics training of undergraduate medical students at South African universities. S Afr Fam Pract. 2009;51(2):158–61. 10.1080/20786204.2009.10873833.

[9] Naing C, Wai VN, Durham J, Whittaker MA, Win NN, Aung K, Mak JW. A systematic review and meta-analysis of medical students’ perspectives on the engagement in research. Medicine (Baltimore). 2015;94(28):e1089. 10.1097/MD.0000000000001089. 26181541 10.1097/MD.0000000000001089 PMC4617066

[10] Nel D, Burman RJ, Hoffman R, Randera-Rees S. The attitudes of medical students to research. S Afr Med J. 2014;104(1):33–6. 10.7196/samj.7058. 10.7196/samj.7058 24388084

[11] Mahomed S, Ross A, Van Wyk J. Training and assessing undergraduate medical students’ research: learning, engagement and experiences of students and staff. Afr J Prim Health Care Fam Med. 2021;13(1):a2559. 10.4102/phcfm.v13i1.2559. 10.4102/phcfm.v13i1.2559 PMC7876945 33567848

[12] Zimbardi K, Myatt P. Embedding undergraduate research experiences within the curriculum: a cross-disciplinary study of the key characteristics guiding implementation. Stud High Educ. 2014;39(2):233–50. 10.1080/03075079.2011.651448.

[13] Ommering BW, Van Diepen M, Van Blankenstein FM, De Jong PG, Dekker FW. Twelve tips to offer a short authentic and experiential individual research opportunity to a large group of undergraduate students. Med Teach. 2020;42(10):1128–33. 10.1080/0142159X.2019.1695766. 33073658 10.1080/0142159X.2019.1695766

[14] Mass-Hernández LM, Acevedo-Aguilar LM, Lozada-Martínez ID, Osorio-Agudelo LS, Maya-Betancourth JGEM, Paz-Echeverry OA, Paz-Echeverry MJ, Castillo-Pastuzan HS, Rojas-Pimentel JC, Rahman S. Undergraduate research in medicine: a summary of the evidence on problems, solutions and outcomes. Ann Med Surg. 2022;74:103280. 10.1016/j.amsu.2022.103280. 10.1016/j.amsu.2022.103280 PMC8807964 35127067

[15] Cornett M, Palermo C, Wallace MJ, Diug B, Ward B. A realist review of scholarly experiences in medical education. Med Educ. 2021;55(2):159–66. 10.1111/medu.14362. 32888210 10.1111/medu.14362

[16] Joubert G, Steinberg WJ, Van der Merwe LJ. The selection and inclusion of students as research participants in undergraduate medical student projects at the School of Medicine, University of the Free State, Bloemfontein, South Africa, 2002–2017: an ethical perspective. Afr J Health Prof Educ. 2019Nov 12;11(2):57–62. 10.7196/AJHPE.2019.v11i2.1081.

[17] Morton PG. Helping students turn scholarly projects and papers into publishable articles. J Prof Nurs. 2016;32(2):75–6. 10.1016/j.profnurs.2016.03.001. 27000191 10.1016/j.profnurs.2016.03.001

[18] Urrunaga-Pastor D, Alarcon-Ruiz CA, Heredia P, Huapaya-Huertas O, Toro-Huamanchumo CJ, Acevedo-Villar T, Arestegui-Sánchez LJ, Taype-Rondan A, Mayta-Tristán P. The scientific production of medical students in Lima, Peru. Heliyon. 2020;6(3):e03542. 10.1016/j.heliyon.2020.e03542. 32215326 10.1016/j.heliyon.2020.e03542 PMC7090346

[19] Bonilla-Escobar FJ, Bonilla-Velez J, Tobón-García D, Ángel-Isaza AM. Medical student researchers in Colombia and associated factors with publication: a cross-sectional study. BMC Med Educ. 2017;17(1):254. 10.1186/s12909-017-1087-9. 29246229 10.1186/s12909-017-1087-9 PMC5732498

[20] Den Bakker CR, Ommering BW, van Leeuwen TN, Dekker FW, De Beaufort AJ. Assessing publication rates from medical students’ mandatory research projects in the Netherlands: a follow-up study of 10 cohorts of medical students. BMJ Open. 2022;12(4):e056053. 10.1136/bmjopen-2021-056053. 10.1136/bmjopen-2021-056053 PMC8981330 35379628

[21] Al-Busaidi IS, Alamri Y. Publication rates and characteristics of undergraduate medical theses in New Zealand. N Z Med J. 2016;129(1442):46–51. 27657158

[22] Joubert G. Research by pre-clinical undergraduate medical students. Med Educ. 2006;40(5):470–1. 10.1111/j.1365-2929.2006.02435.x. 10.1111/j.1365-2929.2006.02435.x 16635140

